# Cross-national comparison of socioeconomic inequalities in obesity in the United States and Canada

**DOI:** 10.1186/s12939-015-0251-2

**Published:** 2015-10-31

**Authors:** Arjumand Siddiqi, Rashida Brown, Quynh C. Nguyen, Rachel Loopstra, Ichiro Kawachi

**Affiliations:** Dalla Lana School of Public Health, University of Toronto, 155 College Street, Room 566, Toronto, ON M5T 3M7 Canada; Department of Health Behavior, Gillings School of Global Public Health, University of North Carolina, Chapel Hill, Chapel Hill, NC US; Division of Epidemiology, School of Public Health, University of California, Berkeley, 101 Haviland Hall, Berkeley, CA 94720-7358 US; Department of Health Promotion and Education, College of Health, University of Utah, 1901 E. So. Campus Drive, #2120, Salt Lake City, UT 84112 US; Department of Sociology, University of Oxford, Manor Road Building, Manor Road, Oxford, OX1 3UQ UK; Department of Society, Human Development, and Health, Harvard T.H. Chan School of Public Health, 677 Huntington Avenue, Kresge Building 7th Floor, Boston, MA 02115 US

**Keywords:** Socioeconomic status, Health inequalities, Obesity, Canada, United States

## Abstract

**Introduction:**

Prior cross-national studies of socioeconomic inequalities in obesity have only compared summary indices of inequality but not specific, policy-relevant dimensions of inequality: (a) shape of the socioeconomic gradient in obesity, (b) magnitude of differentials in obesity across socioeconomic levels and, (c) level of obesity at any given socioeconomic level. We use unique data on two highly comparable societies – U.S. and Canada - to contrast each of these inequality dimensions.

**Methods:**

Data came from the 2002/2003 Joint Canada/U.S. Survey of Health. We calculated adjusted prevalence ratios (APRs) for obesity (compared to normal weight) by income quintile and education group separately for both nations and, between Canadians and Americans in the same income or education group.

**Results:**

In the U.S., every socioeconomic group except the college educated had significant excess prevalence of obesity. By contrast in Canada, only those with less than high school were worse off, suggesting that the shape of the socioeconomic gradient differs in the two countries. U.S. differentials between socioeconomic levels were also larger than in Canada (e.g., PR quintile 1 compared to quintile 5 was 1.82 in the U.S. [95 % CI: 1.52-2.19] but 1.45 in Canada [95 % CI: 1.10-1.91]). At the lower end of the socioeconomic gradient, obesity was more prevalent in the U.S. than in Canada.

**Conclusions:**

Our results suggest there is variation between U.S. and Canada in different dimensions of socioeconomic inequalities in obesity. Future research should examine a broader set of nations and test whether specific policies or environmental exposures can explain these differences.

## Introduction

Obesity is a determinant of a number of conditions that affect well-being and quality of life including cardiovascular disease, diabetes, depression, cancer, and functional limitations [1, 2]. In turn, a variety of mechanisms – including intrauterine and early life nutritional exposures, diet quality, differences in the built environment and exposure to psychosocial stress [1, 3–8] – have been proposed to account for the risk of obesity. Socioeconomic status (SES) is positioned further upstream of each of these mechanisms; socioeconomic status is thus considered to be a ‘fundamental cause’ of these mechanisms [9–12].

Much of the research in this area has come from the United States where the evidence suggests that, along with the overall rise in prevalence of obesity among all socioeconomic groups, there have been persistent inequalities between socioeconomic groups [7, 8, 13]. A growing body of work has now documented that socioeconomic inequalities in obesity are also present in a range of other countries but the magnitude of inequalities varies considerably between them [1, 2, 14–16].

On their face, these societal-level findings lend credence to the hypothesis that societal-level exposures –e.g. policies and other environmental exposures– may be a ‘cause of the cause’ with respect to socioeconomic inequalities in obesity [12]. However, the methods employed by these prior studies make it challenging to generate hypotheses about the specific policies that may be operating to mitigate obesity inequalities in some societies but exacerbate them in others. Most notably, prior studies have relied on the Relative or Slope Indices of Inequality that were first used to understand socioeconomic inequalities in general morbidity [17]. These indices provide a global sense of the difference between socioeconomic groups, but, in the process, these indices tend to obscure specific dimensions of inequalities between particular socioeconomic groups that can help to shed light on the processes that generate the inequalities.

One important dimension of socioeconomic inequality is the *shape* of the gradient; viz., do obesity inequalities extend throughout the socioeconomic spectrum, or are they limited to specific groups? Comparing societal differences in the shape of the SES gradient can help to raise hypotheses about the extent to which diffuse versus localized policies may be warranted for reducing obesity inequalities.

A second key dimension is the magnitude of inequalities between the top to bottom, i.e. how large is the excess prevalence of obesity comparing the highest versus lowest points of the SES spectrum?

A final consideration is the absolute prevalence of obesity within each specific SES group, i.e. is the prevalence of obesity comparable within SES groups across different societies? For example, are low income Canadians just as likely to be obese as low income Americans? How about at the top of the income distribution? A major challenge in empirically evaluating these questions has been the lack of comparable quality information from more than one country that can be merged into, a single dataset. The lack of such data has meant that direct cross-country comparisons remain exceptionally scarce.

In the current paper, we draw on the Joint Canada/United States Survey of Health (JCUSH), a unique source of data that permits examination of all three aforementioned dimensions of inequality and, does so for a pair of countries that offer a strong comparison for generating hypotheses regarding the policies that may influence health and health inequalities, because of the similarities in their populations and political and economic trajectories [18, 19].

## Methods

### Study population

JCUSH is a one-time (2002/2003), cross-sectional, telephone-based survey conducted jointly by Statistics Canada and the United States National Center for Health Statistics. JCUSH is the only known publicly accessible data source that makes it possible to directly compare individuals within and between high-income societies and thus, to assess socioeconomic inequalities in obesity within and across these societies. Nationally representative samples were collected in both countries with very similar protocols. In each country, the target population included adults aged 18 or older residing in households with a landline telephone. The sampling frame excluded institutionalized populations, full-time members of the military, and residents of the US territories and three Canadian territories. The population in the US was stratified by four geographic regions and, in Canada, by province. Within each regional or provincial stratum, the sample collected was proportional to its population size. In each stratum, random digit dialing was used to contact individuals. In the US, Computer-Assisted Telephone Interviews (CATI) were given in English and Spanish, while in Canada, they were given in English and French. Response rates were 50.2 % for the United States and 65.5 % for Canada. Our final analytic sample was restricted to normal weight and obese individuals only and consisted of 3346 residents of the United States and 2221 residents of Canada. Survey weights minimized responses bias by population-weighting respondents by their age, sex and race/ethnicity (in the U.S.) and age, sex and region (in Canada).

### Variable definitions – dependent variable

JCUSH contains *Body Mass Index (BMI)* data based on self-reported height and weight. We compared those in the Obese range (BMI ≥ 30.0 kg/m^2^) to those in the Normal Weight range (18.0 ≤ BMI < 25.0 kg/m^2^). Pregnant women were excluded from our analyses.

### Variable definitions – main independent variables

We used two main measures of SES. *Income* was measured using annual household income quintiles. Annual household income was composed of income from all sources including wages, income from self-employment, dividends and interest, workers’ compensation, retirement pensions, social security, and other sources. Household income was adjusted for household size by dividing by the square root of the number of family members. Household incomes were then categorized into quintiles, thus providing a measure of one’s income relative to others in the sample (and thus in society), rather than a measure of one’s absolute income [20]. Quintiles were calculated separately for the United States and Canadian samples. However, the U.S. dollar amounts corresponding to the income quintiles are relatively comparable between the United States and Canada (adjusted for 2001 purchasing power parity). The greatest difference occurred for the top (fifth) quintile (Table 1). The lower bound of the top income quintile is $89,000 in the United States and $81,000 in Canada. As is the case for many surveys, data on annual family income quintiles was missing at a substantial rate (21 %).

**Table 1:** Prevalence of obesity by income and education, joint Canada-United States survey of health, 2002/03

	All			Women		Men	
			Obese		Obese		Obese
	N^a^	$ (Lower bound)^b^	%	N^a^	%	N^a^	%
United States	5067		21.1**	2859	22.1**	2208	20.1
Canada	3404		15.7	1809	13.3	1595	18.2
Inequities by Income^b^							
United States							
First quintile	1115	$0	26.3	727	28.9	388	22.6
2nd quintile	1082	$24,766	22.2	652	25.4	430	18.6
3rd quintile	878	$39,910	19.2	496	17.6	382	20.9
4th quintile	981	$59,540	19.9	514	19.4	467	20.5
5th quintile (ref.)	1011	$89,183	17.1	470	15.5	541	18.3
Canada							
First quintile	833	$0	17.6	512	15.5	321	20.4
2nd quintile	736	$25,199	16.2	403	14.8	333	17.8
3rd quintile	621	$38,855	16.0	338	12.8	283	19.6
4th quintile	638	$54,167	15.3	311	11.3	327	18.8
5th quintile (ref.)	576	$81,440	13.2	245	10.4	331	14.9
Inequities by education							
United States							
Less than high school	542		29.9*	319	33.2**	223	25.1
High school	1685		23.6**	969	23.6	716	23.7
Technical/trade degree	670		23.3	374	23.3	296	23.4
University/college (ref.)	1765		16.9	899	17.7	866	16.1
Canada							
Less than high school	709		19.2	343	15.8	366	22.3
High school	929		15.4	477	14.1	452	16.6
Technical/trade degree	701		15.5	393	14.6	308	16.6
University/college (ref.)	883		13.9	456	12.8	427	15.1

Percents weighted to the U.S. population as determined from the October 2002 Current Population Survey and weighted to Canadian population as determined from the 1996 Census

^a^Calculated from full sample of Americans or Canadians without requiring valid information on income or education

^b^U.S. dollars adjusted for 2001 purchasing power parity (PPP) from Statistics Canada

*p < 0.10; **p < 0.05 comparing U.S. to Canada. Chi-square statistics were used to assess statistical significance of comparisons between the U.S. and Canada for each SES category

*Education* was a four category variable (less than high school, high school or equivalent, technical/vocational degree/certificate, university or college degree). Education was missing for 3 % of the overall sample.

While multidimensional measures of employment and occupational status were not available, we additionally tested the binary inequality between those whom were employed and those whom were unemployed (but actively looking for work).

We performed imputation for missing income data (but, not for education, where the low level of missing data falls below the threshold for which imputation is suggested). Under a missing-at-random assumption, multiple imputation may allow for more precise and less biased estimates than a complete case analysis [21]. We utilized Stata MP 13 (StataCorp LP, College Station, TX) to implement a procedure called Multiple Imputation by Chained Equation (MICE) which produces imputed datasets by utilizing a series of imputation models, one model for each variable with missing data [22]. We adhered to recommendations of 20 imputations for 10 % to 30 % missing data [23]. Rubin’s rule was used to combine estimates across imputed datasets [22, 24]. Income data was imputed using covariate data (on education, age, foreign-birth, race, marital status, health insurance, *and* BMI). Similarly, missing data on other variables were imputed using the values of non-missing variables. Results with multiple imputed data were qualitatively very similar and suggested the same conclusions as results dropping observations with missing data. We present analyses of income inequalities based on imputed data.

### Variable definitions – covariates

*Race/Ethnicity* was self-reported and coded as ‘white’ or ‘non-white.’ Finer racial categorizations were not available for the Canadian sample. *Marital Status* was dichotomized as married/living common-law/living with a partner versus single/divorced/separated/widowed. Being *foreign-born* was differentiated from native-born. *Health Insurance* status was asked only for the United States sample, since all Canadians are insured as a matter of public policy. U.S. respondents who reported having any type of insurance (public or private) were coded as ‘insured’ while those reporting no insurance were coded as ‘uninsured.’

### Statistical analyses

Statistical analyses were performed using SUDAAN 9.0 (Research Triangle Institute, Research Triangle Park, NC) to account for the complex survey design and to account for post-stratification adjustment weights. Prevalence of obesity were calculated for the US and Canada (Table 1). Chi-square statistics were used to assess statistical significance of comparisons between the U.S. and Canada for each SES category.

In order to assess within-country socioeconomic inequalities, we used log binomial regression to calculate for each country separately, adjusted prevalence ratios (APRs) of obesity compared to the referent category of normal weight, at each quintile of income and each level of education. All models controlled for age, foreign-birth, race, marital status and, health insurance (U.S. only). Analyses were conducted on the whole sample and in sex-specific strata (Table 2). A shortcoming of a bi-national comparison is the lack of statistical power to conduct rigorous empirical tests of differences between the two nations in within-nation socioeconomic inequalities [25, 26]. Instead, these differences can only be descriptively assessed. However, the lack of previous cross-national assessment of differences in dimensions of socioeconomic inequality and lack of publicly available data for a larger set of comparable high-income nations still renders descriptive assessment a critical part of accumulating evidence on cross-national differences in dimensions of inequalities. On the other hand, because of the uniqueness of this data, our analyses were able to directly calculate prevalence ratios in order to compare whether the same socioeconomic position conferred the same (or different) risk of obesity across countries (Canada was the referent category) (Table 3).

**Table 2:** Prevalence ratios of obesity (Compared to Normal Weight) through the socioeconomic distributions of each country, joint Canada-United States survey of health, 2002/03

	All	Among women only	Among men only
	APR^a^ (95 % CI)	APR^a^ (95 % CI)	APR^a^ (95 % CI)
Inequities by Income			
United States			
First quintile (lowest)	**1.82 (1.52 - 2.19)**	**2.17 (1.66 - 2.84)**	**1.52 (1.17 - 1.98)**
2nd quintile	**1.54 (1.28 - 1.85)**	**1.78 (1.36 - 2.33)**	**1.35 (1.04 - 1.77)**
3rd quintile	**1.29 (1.06 - 1.58)**	**1.35 (1.01 - 1.81)**	1.28 (0.97 - 1.67)
4th quintile	**1.22 (1.01 - 1.48)**	1.29 (0.97 - 1.71)	1.16 (0.90 - 1.48)
5th quintile	(Ref)	(Ref)	(Ref)
Canada			
First quintile (lowest)	**1.45 (1.10 - 1.91)**	**1.79 (1.13 - 2.82)**	1.29 (0.91 - 1.83)
2nd quintile	**1.46 (1.12 - 1.91)**	**1.77 (1.12 - 2.79)**	1.32 (0.95 - 1.82)
3rd quintile	1.24 (0.94 - 1.63)	1.46 (0.91 - 2.33)	1.21 (0.87 - 1.69)
4th quintile	1.12 (0.84 - 1.49)	1.06 (0.64 - 1.78)	1.22 (0.88 - 1.68)
5th quintile	(Ref)	(Ref)	(Ref)
Inequities by education			
United States			
< High school	**2.00 (1.68 - 2.38)**	**2.39 (1.90 - 3.01)**	**1.63 (1.25 - 2.13)**
High school	**1.46 (1.27 - 1.68)**	**1.55 (1.28 - 1.88)**	**1.42 (1.16 - 1.73)**
Technical/trade	**1.42 (1.19 - 1.69)**	**1.53 (1.20 - 1.96)**	**1.32 (1.01 - 1.71)**
University/college	(Ref)	(Ref)	(Ref)
Canada			
< High school	**1.59 (1.25 - 2.02)**	**1.48 (1.00 - 2.18)**	**1.54 (1.16 - 2.06)**
High school	1.24 (0.98 - 1.55)	1.32 (0.93 - 1.87)	1.18 (0.88 - 1.57)
Technical/trade	1.13 (0.88 - 1.46)	1.26 (0.88 - 1.82)	1.08 (0.78 - 1.50)
University/college	(Ref)	(Ref)	(Ref)

*APR* Adjusted prevalence ratios, *Uni.* University

Analyses among all: Income - 3346 Americans, 2221 Canadians; Education – 3021 Americans, 2087 Canadians

Analyses among women: Income - 2068 Americans, 1309 Canadians; Education – 1818 Americans, 1205 Canadians

Analyses among men: Income: 1278 Americans, 912 Canadians; Education –1203 Americans, 882 Canadians

Bolded estimates are statistically significant at *p* ≤ 0.05

^a^Referent outcome: normal weight. Odds ratios adjusted for age, age2, foreign-birth, race, marital status, health insurance (only in US) and interaction terms created by multiplying covariates by an indicator for country (i.e., age*United States, age squared*United States, foreign birth*United States, race*United States, marital status*United States)

**Table 3:** Differences in obesity prevalence within income and education groups: joint Canada-United States survey of health, 2002/2003

	All	Among women only	Among men only
	APR^a^ (95 % CI)	APR^a^ (95 % CI)	APR^a^ (95 % CI)
U.S. vs. Canada			
By Income			
First quintile (lowest)	**1.48 (1.22 - 1.81)**	**1.72 (1.33 - 2.22)**	1.22 (0.90 - 1.67)
2nd quintile	1.20 (0.98 - 1.48)	**1.51 (1.13 - 2.01)**	0.96 (0.71 - 1.30)
3rd quintile	1.16 (0.92 - 1.48)	1.28 (0.92 - 1.80)	1.04 (0.76 - 1.43)
4th quintile	1.17 (0.93 - 1.48)	**1.62 (1.11 - 2.36)**	0.94 (0.71 - 1.25)
5th quintile	1.12 (0.86 - 1.46)	1.39 (0.89 - 2.17)	1.05 (0.76 - 1.44)
By education			
< High school	1.24 (0.99 - 1.55)	**1.85 (1.30 - 2.62)**	0.88 (0.64 - 1.21)
High school	**1.36 (1.13 - 1.63)**	**1.56 (1.19 - 2.04)**	1.22 (0.96 - 1.56)
Technical/trade	**1.38 (1.09 - 1.75)**	**1.54 (1.10 - 2.14)**	1.20 (0.86 - 1.68)
University/college	1.04 (0.85 - 1.27)	1.22 (0.91 - 1.65)	0.94 (0.72 - 1.21)

*APR* Adjusted prevalence ratios

Analyses among all: Income - 3346 Americans, 2221 Canadians; Education – 3021 Americans, 2087 Canadians

Analyses among women: Income - 2068 Americans, 1309 Canadians; Education – 1818 Americans, 1205 Canadians

Analyses among men: Income: 1278 Americans, 912 Canadians; Education –1203 Americans, 882 Canadians

Bolded estimates are statistically significant at *p* ≤ 0.05

^a^a Referent outcome: normal weight. Odds ratios adjusted for age, age2, foreign-birth, race, marital status, and health insurance

## Results

### Crude prevalences (Table 1 and Fig. 1)

#### Full sample

The U.S. had a higher overall prevalence of obesity (21.1 %, 15.7 %; *p* < 0.001) than Canada. Remarkably, the prevalence of obesity in the richest quintile in the U.S. (17.1 %) was almost exactly the same as the prevalence in the poorest quintile in Canada (17.6 %). The education differential in obesity was also much greater in the U.S. (a difference of 13.0 % between those most and least educated) than in Canada (where the difference was 5.3 % between the top and bottom). While in the United States, all but the university/college educated group had prevalences above twenty percent, in Canada, no education stratum had a prevalence of obesity that exceeded twenty percent.

**Fig. 1:**
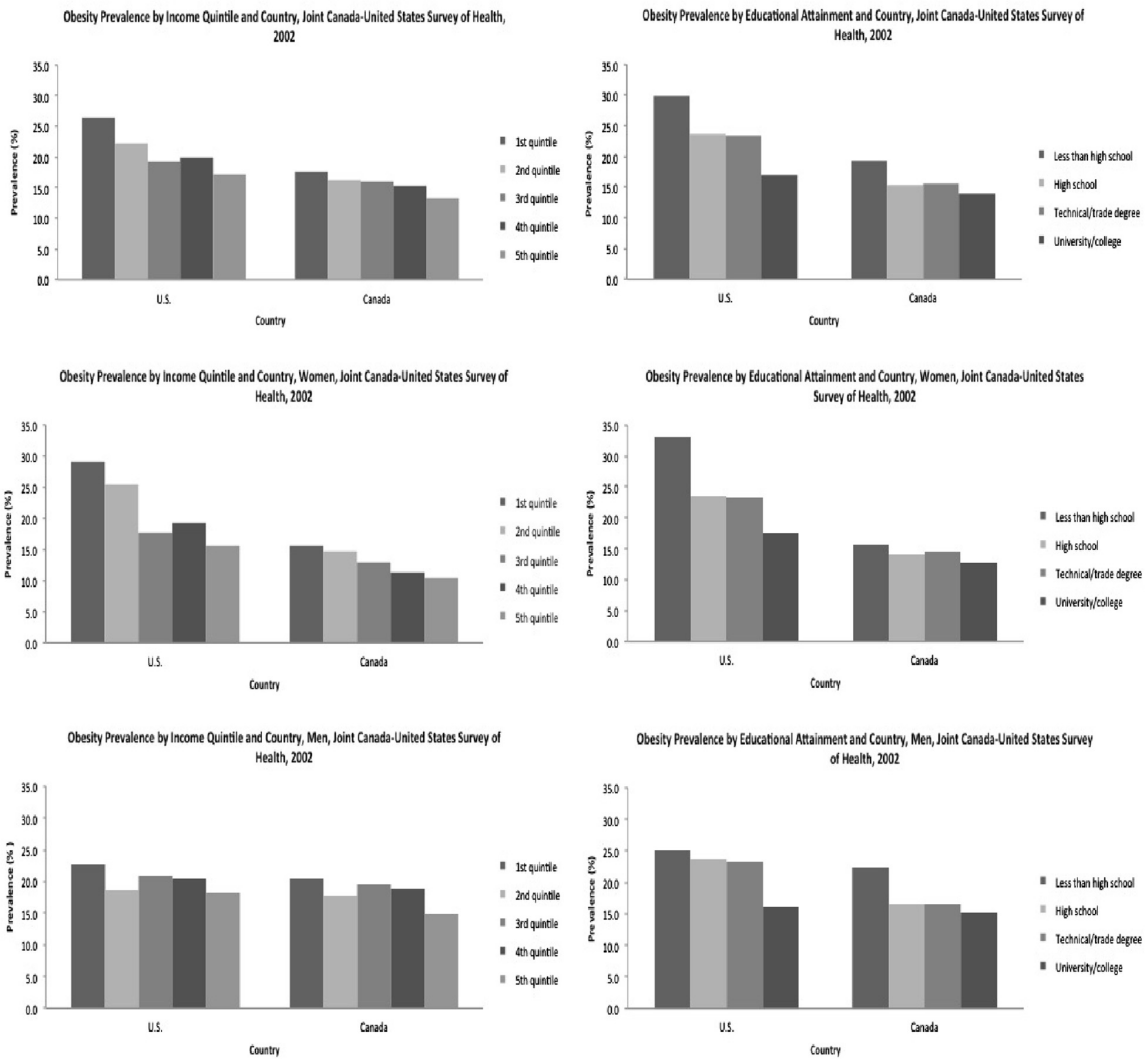
Obesity Prevalence by Country, Socioeconomic Indicator, and Gender

#### Sex-specific strata

US women demonstrated a higher prevalence of obesity compared to women in Canada (22.1 % vs. 13.3 %). No significant cross-national difference in overall prevalence was seen for men. Similar to the main sample, the difference in obesity across income quintiles (13.4 % vs. 5.1 %) and across education levels (15.5 % vs. 3.0) was much larger in the United States than Canada. Amongst men, income and education gradients were less pronounced and more similar across nations than for women. In the full sample, lack of significance appear driven by the lack of significance in men.

### Cross-national differences in the shape of the socioeconomic gradient Table 2

In the full US sample, compared to the richest quintile, there was significant excess adjusted prevalence of obesity in each income quintile, while in Canada, only the first and second quintiles had significantly higher adjusted prevalence of obesity. Tests for downward trend in the US and Canada were significant at *p* ≤ 0.001. Similarly, in the U.S., every educational level had significantly higher adjusted prevalence of obesity relative to the reference group of university/college educated while, in Canada, only those with less than high school education showed an excess obesity prevalence. Tests for downward trend in the two nations were both significant at *p* ≤ 0.001.

Among women, the extent of cross-national difference in the income and education gradients of obesity largely mirrored that of the full sample. Among men, both the first and second quintiles in the United States had significantly higher adjusted prevalence than the fifth quintile while in Canada there were no significant differences in adjusted obesity prevalence across income quintiles. Results for education mirrored the full sample and stratum of women, though effect sizes were smaller. Tests for downward trend in income and education in the U.S. were significant at *p* ≤ 0.001 for both women and men. In Canada, downward trend in income for women was significant at p = 0.001, but not statistically significant for men. For education, downward trend in Canada was significant for both women (*p* < 0.05) and men (*p* < 0.01).

### Cross-national differences in the magnitude of inequalities (Table 2)

In the overall sample, income-based inequalities comparing the poorest to richest quintile were higher in the U.S. compared to Canada (US APR: 1.82, 95 % CI: 1.52-2.19; Canadian APR: 1.45, 95 % CI: 1.10-1.91). This was also the case for the second quintile (US APR: 1.54, 95 % CI: 1.28-1.85; Canadian APR: 1.46, 95 % CI: 1.12-1.91). Cross-national difference in the magnitude of inequality between those with less than high school and those with university/college education was even more pronounced (U.S. APR: 2.00, 95 % CI: 1.68-2.38; Canadian APR: 1.59, 95 % CI: 1.25-2.02).

Among women, cross-national differences in magnitudes were considerably larger than for the full sample. While in the U.S., the adjusted prevalence of obesity at the first quintile was more than twice that of the fifth quintile, in the Canada, the adjusted prevalence at the first quintile was 79 % greater than that of the fifth quintile (U.S. APR: 2.17, 95 % CI: 1.66-2.84; Canadian APR: 1.79, 95 % CI: 1.13-2.82). Inequalities in obesity at the second income quintile were more similar in the two nations. While U.S. women with less than high school education were 2.39 times as likely to be obese (95 % CI: 1.90-3.01), in Canada, they were only 1.48 times as likely to obese (95 % CI: 1.00-2.18).

Employment status demonstrated that, Canadian women had a higher adjusted prevalence ratio, but with only marginal significance (U.S. APR: 1.27, 95 % CI: 1.06, 1.52; Canadian APR: 1.44, 95 % CI: 1.01-2.04). For men, no significant employment-based inequalities in obesity emerged in either nation. (Results not shown).

### Cross-national differences in obesity prevalence within each SES level (Table 3)

Direct cross-national comparisons of individuals in each socioeconomic stratum indicate how much more (or less) each stratum is associated with obesity in the US compared to Canada. In the full sample, compared to those in the bottom income quintile in Canada, those in the lowest income quintile in the U.S. were somewhat more likely to be obese (APR: 1.48, 95 % CI: 1.22-1.81). There were no cross-national differences in other income groups. For education, those in the high school (APR: 1.36, 95 % CI: 1.13-1.63) or technical/trade school (APR: 1.38, 95 % CI: 1.09-1.75) groups had a higher adjusted prevalence of obesity in the United States than in Canada. Compared to the employed in Canada, the employed in the US had higher likelihood of being obese (APR: 1.19, 95 % CI : 1.05-1.35) as did the unemployed in the US compared to the unemployed in Canada (APR: 1.24, 95 % CI: 1.03, 1.49). (Results not shown).

Among women, at nearly every income quintile (but not the third or fifth quintiles) and education level (but not university/college), the U.S. had significantly higher levels of obesity than Canada. Generally, the differences were approximately fifty to sixty percent greater obesity in the United States at any given SES level, but were as high as 85% higher for those with less than high school education. For employment status, full sample results also held for women, but with slightly higher adjusted prevalence ratios (results not shown). Among men, no significant cross-national differences emerged.

## Discussion

This study contrasts three distinct dimensions of socioeconomic inequalities in obesity between two neighboring countries, the United States and Canada: the shape of the socioeconomic gradient in obesity, the magnitude of inequalities contrasting the top and bottom of the socioeconomic spectrum, and the absolute differences in obesity prevalence at the same socioeconomic level. Our study suggests that nations differ not only in overall socioeconomic inequalities of obesity but also across these three dimensions of inequalities

We found that obesity inequalities extended further across socioeconomic groups in the U.S. whereas in Canada, excess obesity prevalence was largely limited to the lowest socioeconomic group. This suggests that the US environment (and accompanying policies) is more “obesogenic” in the sense that only the most privileged groups are able to “escape” its influence. By contrast, in Canada, the shape of obesity disparities suggests that only those at the bottom are vulnerable. For example, the more limited availability in the U.S. compared to Canada of stores from which to purchase healthy foods and, of public parks and recreation facilities, may influence the difference in the range of the socioeconomic gradient implicated in obesity inequalities [27, 28].

We also found that the magnitude of inequalities across the SES spectrum was greater in the U.S. than in Canada. This suggests that whatever accounts for the SES gradients in obesity in each country (whether it be differences in policies or differences in environmental exposures), the “health penalty” paid by socioeconomically disadvantaged groups is greater in the United States than in Canada.

Finally, we found that at lower socioeconomic levels (though not higher ones), there is a higher absolute prevalence of obesity in the US compared to Canada, suggesting that policies targeting the needs of the lowest socioeconomic groups are less effective in the U.S. than policies with similar goals in Canada. For example, aspects of the social safety net designed to buffer the effects of being in the lowest socioeconomic strata (e.g. income transfers and supplementary nutritional assistance to the poor) are less generous in the U.S. than in Canada - though the finding that Canadians in the lowest socioeconomic strata also have higher levels of obesity than mid-strata Canadians suggests that Canadian policies targeted to the lowest socioeconomic rungs are far from sufficient [29, 30].

Taken together, these findings suggest in both countries, policies focused on improving the circumstances of the most disadvantaged groups are warranted. As those who are disadvantaged are unlikely to be effectively influenced by traditional public health interventions focused on individual-level behavior change and which do not tackle root causes [31], Benach and colleagues (2013) have suggested two policy orientations to reduce inequalities in health which warrant consideration: universal policies which provide an additional focus on the worst off (for example, increasing unemployment insurance for the entire population, but with special attention on low-wage occupations) and proportionately universal policies which provide progressively greater benefit as one descends down the socioeconomic spectrum (for example, need-based allocation of community recreational facilities in the context of universal access to these facilities) [32].

At a more basic level, our findings may also be reflective of differences in the degree of income inequality between the two nations. While for sample size reasons, our study was able only to test differences across and between quintile groups, our data and the broader population-level estimates suggest that there are significantly higher levels of income inequality in the United States than in Canada [29, 30]. For the time frame in which the data for our study were collected, estimates from the Organization for Economic Cooperation and Development (OECD) suggest that the Gini coefficient of income inequality averaged 0.375 in the United States and 0.317 in Canada [33]. While cross-national differences in obesity were not observed across the fifth income quintile, it is possible that the lower income quintiles in the United States are more compromised than the lower income quintiles in Canada due to the larger distance to those at the highest end of the income spectrum.

Our findings of larger inequalities in the U.S. compared to Canada are consistent with prior studies, which have also demonstrated more pronounced health inequalities in the US compared to Canada for other outcomes and by other sources of disadvantage (such as race and immigrant status) [18, 29, 34–37]. However, these prior studies did not probe differences in dimensions of inequality. Our findings that inequalities were stronger in women is also consistent with prior findings [38]. These results suggest many different processes may be at play – from work-life/home-life roles, to gendered weight norms. Such phenomena may also be linked to policies such as parental leave policies, though no specific hypotheses have yet been forwarded in this regard [39].

Our study is not without limitations. First, our data are from 2002/2003, the only year in which this survey was conducted, and therefore it is unclear the extent to which our results might be replicated in present day. Second, we were able only to assess cross-sectional associations between socioeconomic indicators and BMI, which prevents us from establishing temporal order between income or education and BMI. Third, our study relied on self-reported rather than laboratory-measured BMI. The biases of self-reported BMI are quite well understood. Men over-estimate their height and women under-estimate their weight – both result in lower reported BMI compared to measured BMI. However, validation studies have suggested that the degree of bias is small (three to nine percent) and similar in Canada and the United States, thus we do not believe that self-reports introduce substantial bias in our cross-national comparative study [40]. Moreover, the consistency of our results with prior self-reported survey data provides further confidence that JCUSH data are not severely skewed [13, 40–44]. Fourth, the sampling frame of the study was based on individuals with landline telephones and thus incurred bias by excluding those without landlines. However, prior estimates suggest that the proportions, though growing, are still quite small (1.8% in Canada and 4.4% in the United States [45, 46]). Thus, it is unlikely that the sampling frame induced large bias. Because those without landline telephones are likely to be in the lowest income quintile, and bias resulting from sampling is likely to have resulted in a slight underestimation of the degree of socioeconomic inequality in BMI and, thus, our paper represents conservative estimates of these inequalities. Finally, though our study makes important contributions to inference across an important geopolitical level – the country – we are unable to account for how inequalities differ across smaller (subnational) scales of geographies such as provinces or states.

Overall, our study highlights the potential utility of examining cross-national differences in health inequalities through the use of multiple metrics. Such a comprehensive approach to describing SES gradients is needed to understand both the origins of inequalities as well as potential solutions. Cross-national comparative research on inequalities has been hampered by the use of restricted measures to describe SES gradients. Further research should explore more robust means of describing and comparing cross-national differences.
